# Non-motor symptoms burden in motor-fluctuating patients with Parkinson’s disease may be alleviated by safinamide: the VALE-SAFI study

**DOI:** 10.1007/s00702-022-02538-w

**Published:** 2022-09-07

**Authors:** Claudia De Masi, Claudio Liguori, Matteo Spanetta, Mariana Fernandes, Rocco Cerroni, Elena Garasto, Mariangela Pierantozzi, Nicola Biagio Mercuri, Alessandro Stefani

**Affiliations:** 1 Neurology Unit, University Hospital of Rome “Tor Vergata”, Rome, Italy; 2 Department of Systems Medicine, University of Rome “Tor Vergata”, Rome, Italy; 3 UOSD Parkinson’s Disease Centre, Department of Systems Medicine, University of Rome “Tor Vergata”, Rome, Italy; 4 IRCCS Santa Lucia Foundation, Rome, Italy

**Keywords:** Pain, Quality of life, Sleep, Daytime sleepiness, Movement disorder

## Abstract

Parkinson’s disease (PD) is characterized by motor symptoms often experienced in concomitance with non-motor symptoms (NMS), such as depression, apathy, pain, sleep disorders, and urinary dysfunction. The present study aimed to explore the effect of safinamide treatment on NMS and quality of life in motor-fluctuating PD patients. VALE-SAFI is an observational single-centre study performed in fluctuating PD patients starting safinamide treatment and followed for 6 months. The effects of safinamide on NMS, sleep, fatigue, depression and pain were assessed through validated sales. Changes in the scales from baseline to the 6-month follow-up visit were analysed. 60 PD patients (66.67% males) were enrolled at baseline, and 45 patients completed the 6-month follow-up. PD patients improved motor symptoms at follow-up, with the significant reduction of motor fluctuations. The global score of the NMS Scale significantly decreased between baseline and the follow-up. Regarding pain domains, patients reported a significant improvement in discolouration and oedema/swelling. Further, a significant improvement was observed from baseline to follow-up in sleep quality measured through the Pittsburgh Sleep Quality Index, while no changes were documented in daytime sleepiness. No differences were found in depression and fatigue between baseline and follow-up. Finally, the patient’s perception of the impact of PD on functioning and well-being decreased from baseline to follow-up. The present findings confirmed the beneficial effect of safinamide on both motor and non-motor symptoms, also improving the quality of life of PD patients. Furthermore, these data support the positive effects of safinamide on pain and mood, as well as on sleep quality and continuity.

## Introduction

Motor symptoms such as bradykinesia, rigidity, postural instability and tremor are the main target of treatment in patients with Parkinson’s disease (PD). Nonetheless, these motor symptoms are often experienced in concomitance with non-motor symptoms (NMS), which include depression, psychosis, apathy, pain, sleep disorders, and urinary dysfunction (Poewe et al. 2017; Schapira et al. 2017; Church 2021). NMS have proven to be present as intrinsic events of the disease, but may also be exacerbated by the dopaminergic treatment (Zis et al. 2015; De Micco et al. 2021). Moreover, the presence of NMS in motor-fluctuating PD patients is consistently high, as recently documented (Fernandes et al. 2021). The frequency of NMS increases with the progression of the disease, in particular when motor fluctuations appear and show a strong negative impact on PD patient well-being and quality of life (Martinez-Martin et al. 2011; Antonini et al. 2012). Despite their clinical relevance, NMS are often under-recognized, and even when are identified through clinicians’ assessment or patients and/or caregivers reports, the range of treatment options is restricted (De Micco et al. 2021). This is probably due to the unclear pathophysiology of NMS, which seems to be related to dysfunction of both the dopaminergic and non-dopaminergic systems (Schapira et al. 2017).

Safinamide is a monoamine oxidase B inhibitors (iMAO-B) currently used as an add-on treatment to a stable dose of Levo-Dopa (LD) in motor-fluctuating PD patients (Mancini et al. 2018; García et al. 2021b). In the last years, safinamide become a useful treatment for both motor and non-motor symptoms in PD patients. Different studies demonstrated the beneficial effects of safinamide on NMS (Liguori et al. 2018a, b; Cattaneo et al. 2020; Geroin et al. 2020; Grigoriou et al. 2021), however, it is important to note that most of these studies had a retrospective design, included small groups of heterogeneous patients, or counted PD patients treated with safinamide in monotherapy. Therefore, the present observational single-centre study aimed to better understand the impact of safinamide treatment on motor impairment and NMS, namely fatigue, sleep depression and pain, as well as on the quality of life in motor-fluctuating PD patients.

## Methods

### Study design

For this observational single-centre study, patients affected by idiopathic PD diagnosed according to the UK Parkinson’s Disease Society Brain Bank criteria were enrolled between September 2018 and March 2020 at the UOSD Parkinson owing to the Neurology Clinic of the University Hospital of Rome “Tor Vergata”. All PD patients included in the study started treatment with safinamide according to clinical practice and guidelines. In particular, the starting administered dose of safinamide was 50 mg/day in the morning for two weeks and then increased to 100 mg/day from the third week of treatment.

The inclusion criteria were the following: (1) adult male and female patients (aged > 18 years); (2) patients able of giving signed informed consent; (3) patients on stable LD treatment, with or without a COMT inhibitor, and/or on a stable dose of dopamine-agonist for at least 4 weeks before the baseline visit; and (4) patients that would be treated with safinamide as an add-on therapy independently of this study. The exclusion criteria were: (1) patients enrolled in other studies and (2) patients in therapy with iMAO-B. In these patients, a washout period of at least 2 weeks was performed to include patients in the present study.

Patients with PD underwent a neurological evaluation and completed self-reported scales at baseline and after 6 months of treatment with safinamide.

### Measures

The Non-Motor Symptoms Scale (NMSS) includes 30 items that assess a wide range of non-motor symptoms grouped in the following 9 domains: cardiovascular (2 items), sleep/fatigue (4 items), mood/cognition (6 items), perceptual problems/hallucinations (3 items), attention/memory (3 items), gastrointestinal tract (3 items); urinary (3 items), sexual function (2 items) and miscellaneous (4 items) (Chaudhuri et al. 2007; Cova et al. 2017).

PD patient’s quality of life was measured with the 39-Item Parkinson's Disease Questionnaire (PDQ-39), which is used to evaluate how often PD patients experience difficulties across 8 dimensions of daily living: mobility (10 items), activities of daily living (6 items), emotional well-being (6 items), stigma (4 items), social support (3 items), cognition (4 items), communication (3 items) and bodily discomfort (3 items) (Jenkinson et al. 1997; Galeoto et al. 2018).

Sleep quality was assessed through the Pittsburgh Sleep Quality Index (PSQI), which is based on 19 self-questions that combined form 7 component scores: subjective sleep quality, sleep latency, sleep duration, habitual sleep efficiency, sleep disturbances, use of sleeping medication, and daytime dysfunction (Buysse et al. 1989; Curcio et al. 2013). Moreover, excessive daytime sleepiness was evaluated through the Epworth Sleepiness Scale (ESS), which help to evaluate patient propensity to doze or fall asleep during 8 common daily activities (Johns 1991; Vignatelli et al. 2003).

Depression symptoms was measured with the Beck Depression Inventory-II (BDI-II), which is a 21-item self-report rating inventory that measures characteristic attitudes and symptoms of depression including: increase or decrease in sleep and appetite, agitation, concentration difficulty and loss of energy (Beck et al. 1996; Sica and Ghisi 2007).

The burden of pain in the context of PD was assessed through the King’s Parkinson’s Disease Pain Scale (King PD) (Jost et al. 2018). This scale is composed of 7 domains including 14 items: musculoskeletal pain (1 item); chronic pain (2 items); fluctuation-related pain (3 items); nocturnal pain (2 items); oro-facial pain (3 items); discoloration, oedema/swelling (2 items); radicular pain (1 item) (Jost et al. 2018).

The Parkinson’s Disease Fatigue Scale (PDFS-16) was used to measure fatigue which is one of the non-motor symptoms of Parkinson Disease (Brown et al. 2005; Siciliano et al. 2019). This scale allows to determine the presence of fatigue (7 items) and its impact on daily function (9 items) (Brown et al. 2005; Siciliano et al. 2019).

The Unified Parkinson's Disease Rating Scale part III (UPDRS-III) and part IV (UPDRS-IV) were used to perform an accurate motor examination (Fahn and Elton 1987).

Clinical data and scores were collected at baseline (T0) and at 6-month follow-up visit (T1). Moreover, tolerability and adverse events (AEs) were collected throughout the entire study period.

The study was approved by the local ethics committee (Tor Vergata CE 160/18) and followed CONSORT guidelines. All patients gave their written consent to the study.

### Statistical analyses

Data were analysed using the SPSS statistical software package for Windows, release 25.0 (IBM. SPSS—Statistical Package for Social Sciences 2020). Continuous variables are expressed as mean and SD; categorical variables are presented as frequencies and percentages. The changes in primary and secondary outcomes between T0 (baseline) and T1 (follow-up) were also assessed using the Wilcoxon Rank-Sum test for comparing continuous variables. A value of *p* < 0.05 was considered statistically significant.

## Results

### Participants and clinical data

**Fig. 1:**
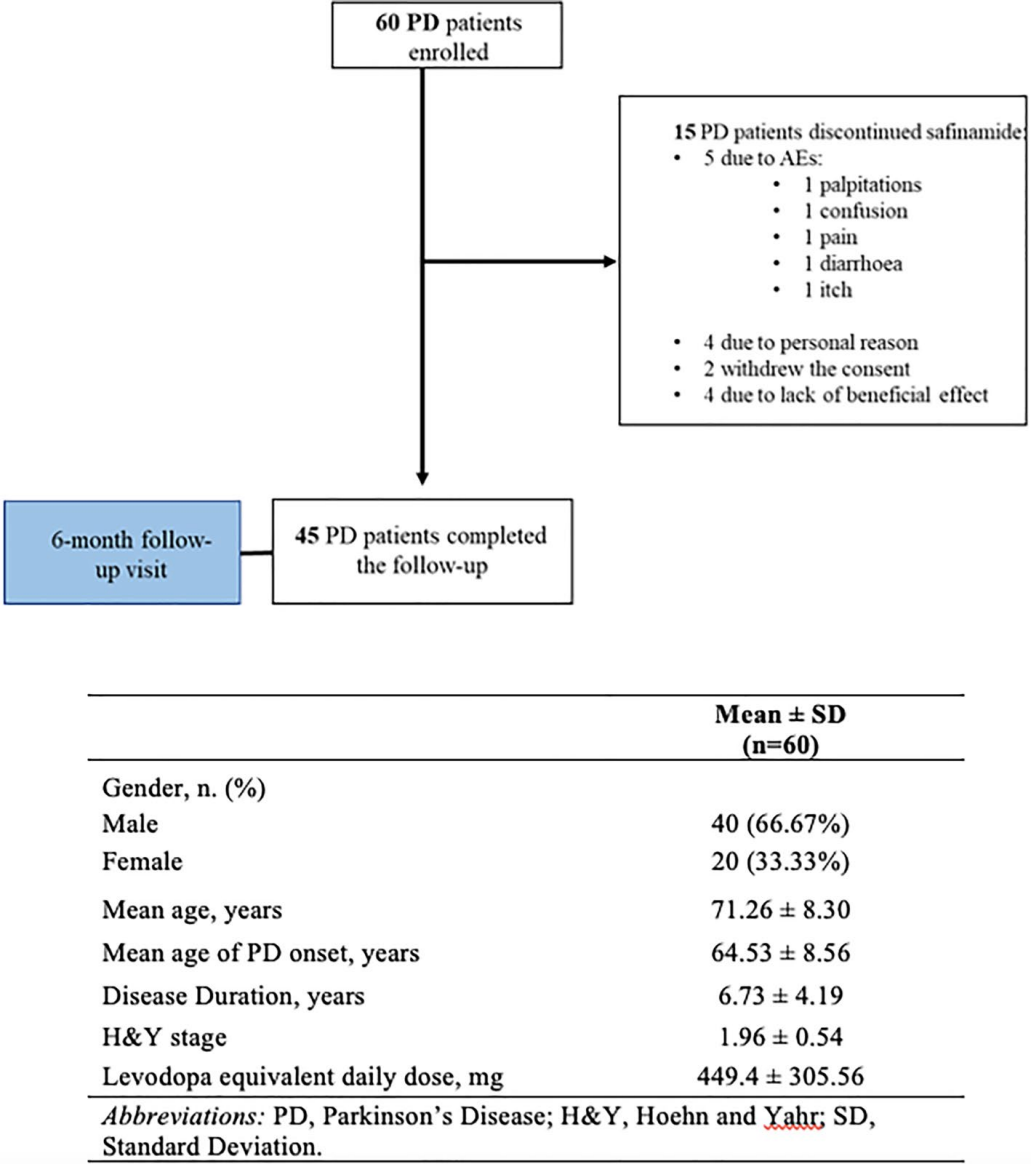
Flow chart of the study and patients’ baseline demographic and clinical characteristics

Sixty patients were enrolled in this study, completed the baseline evaluation and started safinamide treatment. After 6 months, 45 patients completed the follow-up visit and presented a significant reduction of both UPDRS-III and -IV (Table 1). Patients’ baseline demographic and clinical characteristics are presented in Fig. 1.

**Table 1:** Patients’ motor disability, non-motor symptoms, and quality of life

	Baseline Mean ± SD (*n* = 45)	Follow-up Mean ± SD (*n* = 45)	*p* value
Motor disability			
UPDRS-III score	23.73 ± 4.80	21.66 ± 4.50	0.032
UPDRS-IV score	3.00 ± 3.87	1.40 ± 2.83	** < **0.001
Non-motor symptoms			
NMSS Domains			
Cardiovascular	2.73 ± 5.00	2.46 ± 5.00	0.355
Sleep / Fatigue	17.00 ± 15.96	13.26 ± 13.59	0.511
Mood / Cognition	28.66 ± 19.67	26.60 ± 18.48	0.229
Perceptual problems / Hallucinations	1.66 ± 4.36	2.40 ± 4.62	0.433
Attention / Memory	12.20 ± 11.37	10.53 ± 11.26	0.376
Gastrointestinal tract	5.26 ± 5.33	5.46 ± 5.50	0.738
Urinary	16.00 ± 11.53	15.86 ± 10.05	1.000
Sexual function	5.33 ± 8.57	3.33 ± 6.25	0.622
Miscellaneous	10.93 ± 7.84	6.06 ± 5.75	0.004
NMSS Total	99.80 ± 48.75	86.0 ± 40.87	0.035
Quality of life			
PDQ-39 Domains			
Mobility	23.83 ± 21.88	24.17 ± 20.01	0.893
Activities of daily living	17.22 ± 15.22	17.78 ± 15.19	0.685
Emotional well-being	37.78 ± 25.54	33.33 ± 23.23	0.005
Stigma	27.02 ± 30.52	14.58 ± 19.54	** < **0.001
Social Support	8.89 ± 13.92	2.78 ± 5.89	0.002
Cognition	24.17 ± 14.93	26.25 ± 24.01	0.776
Communication	11.67 ± 7.41	7.22 ± 10.60	0.005
Bodily discomfort	33.33 ± 26.29	35.56 ± 32.78	0.530
PDQ-39 Total	23.10 ± 11.44	20.21 ± 13.31	0.012

*UPDRS,* Unified Parkinson's Disease Rating Scale; *NMSS,* Non-Motor Symptoms Scale; *PDQ-39,* 39-Item Parkinson's Disease Questionnaire; *SD,* Standard Deviation

### Global NMS and quality of life data

Regarding the NMS, the global score of the NMS scale significantly decreased between baseline and follow-up. Considering the single-domain analysis of NMS scale, there was only a significant reduction from baseline to follow-up on domain 9, which assesses different aspects of NMS in PD (pain among them).

PD Patients reported a significant decrease in PDQ-39 global score from baseline to follow-up, which indicates an improvement on patients’ quality of life after safinamide treatment. Moreover, at the follow-up visit, a significant change in different domains of the questionnaire was evident, namely emotional well-being, stigma, social support, communication. All NMS and quality of life data are reported in Table 1.

### Depression and fatigue data

Regarding the depressive symptoms assessed by BDI-II, no differences were found between baseline and follow-up in the global score. The mean score of item 17, which measures irritability, showed a significant reduction from baseline to the follow-up visit. Moreover, no differences were evident between baseline and follow-up either in the global or in the sub-item scores of the PDFS-16 scale. All depression and fatigue data are reported in Table 2.

**Table 2:** Depression and fatigue data

	Baseline Mean ± SD (*n* = 45)	Follow-up Mean ± SD (*n* = 45)	*p* value
BDI-II			
1. Sadness	0.73 ± 0.68	0.73 ± 0.78	0.875
2. Pessimism	1.00 ± 1.04	0.66 ± 0.87	0.117
3. Past failure	0.40 ± 0.61	0.26 ± 0.57	0.188
4. Loss of pleasure	0.80 ± 0.54	0.73 ± 0.86	0.223
5. Guilty feelings	0.13 ± 0.34	0.26 ± 0.78	0.902
6. Punishment feelings	0.33 ± 0.79	0.40 ± 0.88	0.917
7. Self-dislike	0.26 ± 0.44	0.26 ± 0.44	1.000
8. Self-criticalness	0.46 ± 0.50	1.00 ± 1.98	0.566
9. Suicidal thoughts/wishes	0.26 ± 0.57	0.20 ± 0.40	0.876
10. Crying	0.53 ± 0.62	0.60 ± 0.88	0.805
11. Agitation	0.80 ± 0.84	0.53 ± 0.81	0.058
12. Loss of interest	0.73 ± 0.86	0.60 ± 0.61	0.718
13. Indecisiveness	0.8 ± 0.75	0.80 ± 0.75	1.000
14. Worthlessness	1.00 ± 1.10	0.66 ± 0.95	0.141
15. Loss of energy	1.00 ± 0.63	1.26 ± 1.00	0.244
16. Changes in sleeping pattern	0.93 ± 0.93	0.93 ± 1.07	0.815
17. Irritability	1.40 ± 0.61	0.86 ± 0.62	** < **0.001
18. Changes in appetite	0.73 ± 0.93	0.46 ± 0.72	0.172
19. Concentration difficulty	0.20 ± 0.54	0.20 ± 0.75	0.364
20. Tiredness or fatigue	1.00 ± 0.97	0.86 ± 1.03	0.437
21. Suicidal thoughts or wishes	0.53 ± 0.89	0.40 ± 0.80	0.443
BDI-II Total	14.06 ± 6.97	12.73 ± 9.8	0.344
PDFS-16			
1. Have to rest during the day	1.93 ± 1.25	2.06 ± 1.25	0.617
2. Life restricted by fatigue	1.93 ± 1.30	1.73 ± 1.19	0.451
3. Tired more quickly than other people	2.26 ± 1.00	2.26 ± 1.19	0.879
4. One of my three worst symptoms	1.20 ± 1.12	1.40 ± 1.26	0.519
5. Feel completely exhausted	1.13 ± 1.21	1.13 ± 0.89	0.705
6. Reluctant to socialize	1.20 ± 1.23	0.93 ± 0.86	0.465
7. Takes longer to get things done	2.20 ± 1.17	1.73 ± 1.19	0.065
8. Feeling of heaviness	1.73 ± 1.13	1.73 ± 1.19	0.940
9. Could do more if not tired	1.86 ± 1.15	1.80 ± 1.12	0.704
10. Everything is an effort	1.53 ± 1.21	1.60 ± 1.15	0.652
11. Tired much of the time	1.53 ± 1.21	1.53 ± 1.03	1.000
12. Totally drained	1.40 ± 1.09	1.40 ± 1.09	1.000
13. Difficult to cope with everyday activities	1.53 ± 1.27	1.53 ± 1.15	0.970
14. Tired even when I have not done anything	1.73 ± 1.45	1.46 ± 1.21	0.408
15. Do less in my day than I would like	1.86 ± 1.21	1.60 ± 1.09	0.226
16. So tired I want to lie down wherever I am	1.26 ± 1.30	1.20 ± 1.23	0.908
PDFS-16 Total	26.33 ± 13.35	25.13 ± 14.12	0.636

*BDI-II,* Beck Depression Inventory-II; *PDFS-16,* Parkinson’s Disease Fatigue Scale; *SD,* Standard Deviation

### Pain data

Regarding pain evaluated through the KPDPS, only a significant result from baseline to follow-up was found, specifically a decrease in discolouration oedema/swelling (domain 6). Pain data are reported in Table 3.

**Table 3:** Pain data

	Baseline Mean ± SD (*n* = 45)	Follow-up Mean ± SD (*n* = 45)	*p* value
KPPS domains			
Musculoskeletal pain	4.26 ± 4.03	3.73 ± 3.75	0.495
Chronic pain	3.06 ± 5.56	2.26 ± 4.23	0.473
Fluctuation-related pain	1.33 ± 4.03	2.80 ± 4.88	0.093
Nocturnal pain	4.60 ± 6.54	4.40 ± 6.48	0.640
Oro-facial pain	0.13 ± 0.50	0.13 ± 0.50	1.000
Discoloration, oedema/swelling	1.73 ± 3.03	0.53 ± 2.01	0.016
Shooting pain/pins & needles	0.73 ± 2.10	0.66 ± 2.04	0.951
KPPSS Total Score	15.86 ± 18.54	14.53 ± 16.18	0.853

*KPPS,* King’s Parkinson’s Disease Pain Scale; *SD,* Standard Deviation

### Sleep and daytime sleepiness data

Finally, a significant reduction of the global score of the PSQI was observed from baseline to follow-up, indicating an increase in sleep quality. Moreover, different components of the PSQI significantly improved between baseline and follow-up (C1, subjective sleep quality; C2, sleep latency; C4, habitual sleep efficiency; C5, sleep disturbances; C7, daytime dysfunction). Considering the excessive daytime sleepiness, no significant changes were evident between baseline and follow-up on ESS scores (Table 4).

**Table 4:** Sleep quality and daytime sleepiness data

	Baseline Mean ± SD (*n* = 45)	Follow-up Mean ± SD (*n* = 45)	*p* value
Sleep quality			
PSQI subscales			
Subjective sleep quality	0.93 ± 1.01	0.51 ± 0.59	** < **0.001
Sleep latency	1.47 ± 1.22	1.31 ± 1.08	0.008
Sleep duration	1.37 ± 0.86	1.22 ± 0.85	0.157
Habitual sleep efficiency	1.47 ± 1.34	0.78 ± 0.80	** < **0.001
Sleep disturbances	1.60 ± 1.16	0.87 ± 0.76	** <** 0.001
Use of sleep medication	0.87 ± 1.04	0.91 ± 0.97	0.317
Daytime dysfunction	0.40 ± 0.72	0.11 ± 0.32	0.002
PSQI global score	8.00 ± 4.87	5.69 ± 3.23	** < **0.001
Daytime sleepiness			
ESS score	7.26 ± 5.25	7.53 ± 5.41	0.770

*PSQI,* Pittsburgh Sleep Quality Index; *ESS,* Epworth Sleepiness Scale; *SD* Standard Deviation

## Discussion

NMS frequently occur in patients with PD, and it has been estimated that their prevalence can reach 90% along with the disease progression. Moreover, PD patients with motor fluctuations can present an increased prevalence of NMS from 80% to more than 90% in patients with high H&Y stages and long disease duration (Fernandes et al. 2021).

The main result of the present study is the significant reduction of NMSS global score between baseline and follow-up, which ameliorated in parallel with the significant improvement of UPDRS-III and -IV scores. These findings confirmed the beneficial effect of safinamide on both motor and non-motor PD symptoms. This significant improvement reflects also the amelioration of patients’ QoL and well-being measured through the PDQ-39. Consistently, several items related to the impact of PD symptoms on QoL improved from baseline to follow-up, thus highlighting the significant effect of safinamide on different aspects of daytime activities, social relations, and emotional well-being.

The significant improvement of domain 9 of NMSS, that measures pain and miscellaneous NMS, was also evident, and sleep quality and continuity significantly improved after safinamide treatment, which did not affect daytime vigilance, since the ESS scores did not change between baseline and follow-up.

NMS increasingly show a critical role in the clinical characterization of patients with PD due to their disabling nature, challenges in the management, and negative impact on patients’ and caregivers’ well-being. Sleep disorders and psychiatric symptoms are the main NMS in motor-fluctuating PD patients, although pain and fatigue commonly affect those patients (Fernandes et al. 2021). There is still no evidence of beneficial effects of dopaminergic treatment on the entire NMS spectrum, possibly since NMS may refer to multi-system networks, involving not only the dopaminergic but also the other monoaminergic or orexinergic, glutamatergic and GABA-ergic systems (Miguelez et al. 2020). Therefore, it has been suggested that the best hypothetical treatment for NMS should present a multi-neurotransmitting system effect.

One of the most recent drugs approved for the treatment of motor-fluctuating PD patients is safinamide, which consists of an orally administered α-aminoamide derivative able to target both dopaminergic and glutaminergic systems through its multiple mechanism of action. In particular, safinamide can act as a selective and reversible iMAO-B and can also block voltage-dependent sodium and calcium channels, with inhibition of dopamine reuptake and modulation of glutamate and GABA release (Bianchi et al. 2019).

Aside from motor symptoms improvement, different recent reports demonstrated a significant effect of safinamide on NMS (Bianchi et al. 2019; Geroin et al. 2020; De Micco et al. 2021; Grigoriou et al. 2021; Peña et al. 2021). The single open-label prospective study on different NMS performed in Spain included fifty PD patients but not all were treated with safinamide as an add-on treatment to LD (García et al. 2021a, b; Labandeira et al. 2021; Santos García et al. 2021). The first published result showed the significant reduction of NMSS global score after 6 months of safinamide treatment in PD patients. Moreover, the items related to sleep and fatigue, mood, apathy, attention and memory, gastrointestinal and urinary symptoms, and pain were significantly reduced at the follow-up (García et al. 2021b). Following this report, other results have been published from this multi-centre study, but separately analysed, documenting an improvement of sleep, daytime sleepiness, depression, and pain (García et al. 2021a; Labandeira et al. 2021; Santos García et al. 2021). Considering the importance of a comprehensive and prospective analysis including an homogeneous large sample of motor-fluctuating PD patients treated by safinamide as add-on treatment to LD, the present study collectively evaluated all these NMS at baseline and after 6 months of stable treatment in a group of sixty motor-fluctuating PD patients.

Pain improvement after safinamide treatment was evident already in the post-hoc analysis of the registrative trials (Cattaneo et al. 2020). Consistently, real-world data confirmed this preliminary observation on pain symptoms also through the use of self-reported questionnaires and scales that assess this specific NMS (Chaudhuri et al. 2007; Cova et al. 2017). In line with this evidence, the present study documented a significant reduction from baseline to follow-up in discolouration oedema/swelling (domain 6 of the KPPS scale) in PD patients treated with safinamide, which was already described in previous studies (Geroin et al. 2020; García et al. 2021a; Grigoriou et al. 2021). Regarding mood, the improvement in irritability, a symptom commonly reported during a depressive episode and a frequent behavioural symptom in PD patients, was evident at follow-up. Further investigations regarding the effect of the drug on irritability and behaviour should be planned to confirm this preliminary data. Finally, a significant effect of safinamide on subjective sleep quality, sleep latency, sleep efficiency, and then on sleep disturbances was observed between baseline and follow-up. This finding concords with previous and preliminary reports about the effects of safinamide on sleep measured either objectively through polysomnographic recording or subjectively with different scales (Liguori et al. 2018a, b; Santos García et al. 2021).

No effects of safinamide treatment were evident on fatigue, although item 7 that measured daytime productivity presented a trend in improvement at follow-up, as well as on daytime sleepiness, which can be considered as a positive indicator since other dopaminergic drugs have been associated with sleep attacks and excessive daytime sleepiness (Hauser et al. 2000; Chaudhuri et al. 2002; O’Suilleabhain and Dewey 2002; Valko et al. 2010; Yeung and Cavanna 2014). Notably, the beneficial effect of safinamide on daytime dysfunction was evident on the PSQI component, with a reduction of this component at follow-up.

In conclusion, the present study confirmed the previous evidence about the beneficial effect of safinamide on NMS in motor-fluctuating PD patients. The mechanism of action of the drug, including not only the dopaminergic effect but also the modulation of glutamatergic and GABA-ergic systems (Morari et al. 2018; Pisanò et al. 2020), may substantiate the clinical evidence achieved in this study. The open-label design and the 6-month follow-up analysis were limitations of this study. Further investigations with a longer follow-up should be planned to test the maintenance of the positive effect on NMS and its correlation with motor symptoms and motor fluctuations.

## Data Availability

The data that support the findings of this study are available from the corresponding author, upon reasonable request.
